# The Effect of Combustion Conditions on Emissions of Elemental Carbon and Organic Carbon and Formation of Secondary Organic Carbon in Simulated Wildland Fires

**DOI:** 10.1021/acsestair.4c00300

**Published:** 2025-09-11

**Authors:** Robert Penland, Steven Flanagan, Luke Ellison, Muhammad Abdurrahman, Chase K. Glenn, Omar El Hajj, Anita Anosike, Kruthika Kumar, Mac A. Callaham, E. Louise Loudermilk, Nakul N. Karle, Ricardo K. Sakai, Adrian Flores, Tilak Hewagam, Charles Ichoku, Joseph O’Brien, Rawad Saleh

**Affiliations:** † School of Environmental, Civil, Agricultural, and Mechanical Engineering, 1355 University of Georgia, Athens, Georgia 30602, United States; ‡ USDA Forest Service Southern Research Station, Athens, Georgia 30602, United States; § 14701 University of Maryland − Baltimore County, Baltimore, Maryland 21250, United States; ∥ Howard University Beltsville Campus, Beltsville, Maryland 20705, United States; ⊥ 53523 NASA Goddard Space Flight Center, Greenbelt, Maryland 20771, United States

**Keywords:** wildfires, prescribed fire, smoke, combustion conditions, fire radiative energy, carbonaceous aerosol, emission factors

## Abstract

We investigated the influence of combustion conditions on emissions of elemental carbon (EC) and organic carbon (OC) and the formation of secondary organic carbon (SOC) in wildland fires. We performed combustion experiments using fuel beds representative of three ecoregions in the Southeastern U.S. and varied the fuel-bed moisture content to simulate either prescribed fires (Rx) or drought-induced wildfires (Wild). We used fire radiative energy normalized by fuel-bed mass (FRE_norm_) as a proxy for combustion conditions. For fuel beds that contained surface fuels only, the higher moisture content in Rx led to lower FRE_norm_ compared to Wild and consequently led to lower EC emissions, but higher OC emissions and SOC formation. For fuel beds that contained duff in addition to surface fuels, duff did not ignite in Rx because of the high moisture content. However, duff ignited in Wild, leading to prolonged smoldering and substantially lower FRE_norm_ in Wild compared to Rx. Consequently, OC emissions and SOC formation were an order of magnitude higher in Wild compared to Rx for the duff-containing fuel beds. These findings indicate that characterizing fuel availability and variability in combustion conditions, which emerges from variability in fuel-bed composition and environmental conditions, is crucial for determining carbonaceous aerosol formation in wildland fires.

## Graphical abstract

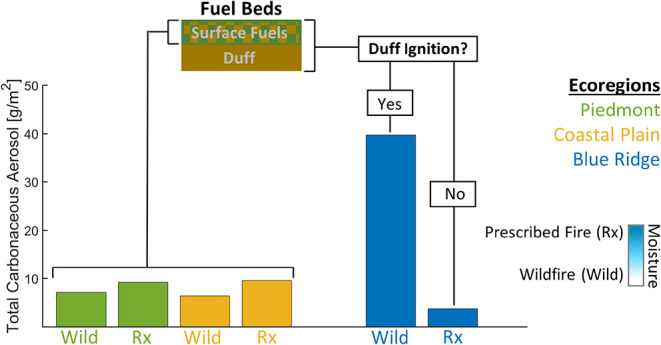

## 1 Introduction

Wildland fires play a crucial role in sustaining the ecological health of many forest types.^1,2^ However, these fires emit smoke, which has significant impacts on public health^3–7^ and the climate system.^8–11^ Wildland fires can occur through accidental or intentional (arson) human ignitions or through lightning ignitions (wildfires), or they can be ignited intentionally for the purpose of forest management (prescribed fires).^2^ Historically, wildland fires in the U.S. were largely controlled by human ignition by indigenous populations, but this trend has shifted dramatically over the past century in part due to fire exclusion practices post Euro-American settlement^12^ leading to significant increase in burned areas consumed by wildfires.^4^ However, there is considerable geographical disparity, where the majority of prescribed fires occur in the Southeastern U.S. and the majority of wildfires occur in the Western U.S.^13^ An important barrier to the use of prescribed fires as tools for ecosystem rejuvenation and preventing large-scale wildfires, however, is the effect of smoke on air quality, both from a regulatory perspective and public perception.^14^ Therefore, improving the understanding of how smoke emissions differ between prescribed fires and wildfires is crucially important to informing the utility of prescribed fires.

Assessing the air-quality impacts of wildland-fire smoke requires estimating the amount of smoke emissions from the fire. The amount of a certain species emitted from a wildland fire can be calculated as^15^

1 Emission=Burned
Area×Fuel Loading×Consumption Fraction×Emission Factor

Despite the apparent simplicity, the determination of each of the terms on the right-hand side of eq 1 is highly complex, leading to a large uncertainty in quantifying smoke emissions from wildland fires.^16–18^ There is a large body of literature that addresses each of the terms in eq 1 and ongoing work continues to do so, with contributions from various fields including forestry, fire science, remote sensing, and atmospheric chemistry. In this paper, we address Consumption Fraction (the fraction of the dry mass of the fuel consumed in a fire) and Emission Factor (the mass of a certain smoke constituent emitted per unit dry mass fuel burned, usually reported in units of g/kg). The picture is further complicated by the chemical evolution of smoke in the atmosphere, an important aspect of which is gas-to-particle conversion of organic species, or secondary organic aerosol (SOA) formation.^19^

Fuel consumption in wildland fires has been actively studied for more than half a century, not only for the purpose of estimating smoke emissions but also because understanding fuel consumption itself is required for supporting prescribed fire programs.^17,20–22^ A central goal of prescribed fires is reduction of hazardous fuels. Therefore, accurate estimates of consumption rates of different components of a fuel bed at different environmental conditions are required to define region-specific conditions appropriate for executing a burn,^21^ often referred to as prescription windows.

Emission factors of smoke constituents have also been extensively studied. Results from various field measurements and laboratory experiments have been compiled in several review/synthesis papers over the past two decades, both from a land-management perspective^17^ and atmospheric-chemistry perspective.^23–26^ Furthermore, recent large-scale collaborative efforts, WE-CAN 2018 (the Western wildfire Experiment for Cloud Chemistry, Aerosol Absorption and Nitrogen 2018) and the NOAA/NASA FIREX-AQ (Fire Influence on Regional to Global Environments and Air Quality),^27^ have deployed state-of-the-science techniques to study wildland-fire smoke, and have reported emission factors of hundreds of smoke constituents from various regions in the U.S.^28–30^

Despite these extensive efforts, there is still a large uncertainty that prohibits accurate representation of fuel consumption and emission factors in emission inventories, as indicated in the most up-to-date reviews.^17,23^ While part of this uncertainty is undoubtedly due to discrepancy between measurement techniques, it is mainly due to true variability across studies, which has been attributed to differences in fuel type and burning conditions.^17,24,30^ The question of whether emission factors are more dependent on fuel type or burning conditions is still not resolved. On the one hand, review/synthesis studies have typically categorized emission factors based on vegetation (land-cover) type or region,^17,23,25^ which is also the basis of how emission factors are implemented within the Fire Inventory from NCAR (FINN).^16^ On the other hand, there is evidence in field measurements that burning conditions can in some cases be more important than fuel type in controlling emissions.^24,28^ Furthermore, previous studies have pointed out discrepancies between laboratory and field measurements of emission factors from the same fuel types, which have been attributed to the fact that the burning conditions in laboratory experiments do not replicate those in the field.^23,24^

Quantifying the relative influence of fuel type and burning conditions on emissions is challenging. First, it is worth noting that the terms “fuel type” and “burning conditions” – and other similar permutations of these terms – do not have formal definitions and can refer to different things in different studies. Fuel type is often broadly defined to mean land-cover or region,^16,17,24^ but has also been used to refer to specific constituents of the fuels consumed in a fire (e.g., litter or wood).^29^ The use of the term burning conditions is even more inconsistent. It has been used to refer to the environmental conditions at which the burn takes place,^23,24^ type of fire (prescribed fire versus wildfire),^17^ or the conditions of the combustion process itself (e.g., smoldering versus flaming).^17,28,29^ Second, setting aside the inconsistencies in definitions, variabilities in fuel type and burning conditions (environmental conditions, or conditions of the combustion process) are not completely orthogonal. Regions with different climate regimes have different vegetation (fuel types) and can also be generally characterized by different environmental conditions (for example humidity), which lead to differences in fuel moisture content and consequently combustion conditions.^21^ Furthermore, for the same environmental conditions, different fuel types can combust differently. For example, forest litter typically exhibits both flaming and smoldering combustion whereas duff combustion is predominantly smoldering.^20,21^ Third, fuel availability (whether the fuel ignites or not) is highly dependent on environmental conditions. For example, surface fuels can be available for combustion during a fire that takes place shortly after rainfall but duff is usually not, whereas after a drought, both surface fuels and duff can be available for combustion.^20,21^ Importantly, the dependence of fuel availability on environmental conditions leads to differences between wildfires and prescribed fires. Even though wildfires can occur at a wide range of environmental conditions, they are often drought-induced and thus feature dry fuels.^20^ Prescribed fires, however, are typically conducted during favorable environmental conditions (prescription window) when the fuels are neither too moist nor too dry,^2,20^ with rare exceptions.^31^

SOA formation in biomass-burning emissions has also been extensively studied both in the field^32–35^ and the laboratory,^36–38^ with wide variation between studies.^14^ In a recent review, Hodshire et al.^19^ attributed the discrepancy between studies to several possible factors, including variability in emissions and chemistry, as well as measurement artifacts and differences in experimental settings and techniques.

This paper presents a systematic investigation of the effects of “fuel type” and “burning conditions” on fuel consumption and the emissions of particulate elemental carbon (EC) and organic carbon (OC), as well as SOA formation potential. Given the ambiguity associated with the terms fuel type and burning conditions described above, it is useful to provide clear definitions of the terminology employed in this paper before stating the specific goals. Instead of fuel type, we use the term “fuel bed” to refer to the forest biomass that can undergo ignition in a wildland fire and “fuel-bed composition” to refer to the proportions of the various fuel constituents within a fuel bed on dry-mass basis. We distinguish between surface fuels, including recently senesced (undecomposed) litter and woody fuels that accumulate on top of the forest floor, and duff, the layer of the forest floor underlying the litter layer, which is composed of partially decomposed organic material.^39,40^ We use the term “combustion conditions” to refer to the characteristics of the combustion process. There are two considerations. First, complete characterization of combustion conditions is not straightforward. However, certain proxies can be used to characterize combustion conditions, the utility of which depends on the application. Proxies can be observations of the fire itself, such as combustion temperature or fire radiative power (FRP),^41,42^ or ratios of key emissions, such as CO_2_/(CO_2_ + CO) (often referred to as the modified combustion efficiency; MCE)^43,44^ or EC/OC.^45^ Second, combustion conditions is not an independent variable, but rather a consequence of fuel-bed characteristics (e.g., composition, mass loading, compactness) and environmental conditions (e.g., wind speed and relative humidity).

The goals of this paper are 2-fold. First, we assess how the variability in fuel bed-composition and environmental conditions impact fuel consumption, EC and OC emissions, and SOA formation. To that end, we performed combustion experiments using fuel beds representative of three different ecoregions in the Southeastern U.S. The variability in fuel-bed composition was reflected in both the makeup of the surface fuels as well as in whether the fuel bed contained duff or not. We considered one variable related to environmental conditions, namely the moisture content. Moisture content is a key differentiator between prescribed fires and drought-induced wildfires^20^ and is highly correlated with fuel consumption, especially for fuel beds that contain duff.^20,21^ Furthermore, difference in moisture content was hypothesized to be the largest contributor to differences in reported emission factors between field measurements and laboratory studies.^24^ Second, we explore the utility of FRP observations for predicting EC and OC emissions and SOA formation, which builds on previous work by Ichoku and co-workers who demonstrated that total particulate matter emissions were correlated with time-integrated FRP (or fire radiative energy, FRE).^42^ This exploration is based on the hypothesis that combustion conditions, captured via FRP measurements as a proxy, can serve as a link between variability in fuel-bed composition and environmental conditions on one hand, and EC and OC emissions and SOA formation on the other. We stress that this paper does not aim to quantify ecoregion-specific or fuel-specific emissions, but the effect of variability in combustion conditions on emissions, as described above. Therefore, while the information on the different ecoregions is retained when presenting the results, the data from all experiments are analyzed and presented together to establish trends with respect to combustion conditions.

## 2 Methods

### 2.1 Overview

The measurements described in the subsequent sections were performed as part of the Georgia Wildland-fire Simulation Experiment (G-WISE), an intensive laboratory campaign that took place in October-November 2022 at the U.S. Forest Service Southern Research Station Prescribed Fire Science Laboratory on the campus of the University of Georgia in Athens, GA. We conducted combustion experiments using fuel beds that were constructed from fuels collected from three ecoregions representative of forests in the Southeastern U.S.:^46^ (1) Piedmont (P), collected from Oconee National Forest, (2) Coastal Plain (CP), collected from Fort Stewart Army Base, and (3) Blue Ridge Mountains (BR), collected from Chattahoochee National Forest. These ecoregions can be broadly mapped to land-cover types in the FINN emissions inventory^16^ (P: Mixed Forests; CP: Woody Savanna; BR: Deciduous Broadleaf Forest). We varied the moisture content of the fuel beds to reflect conditions representative of either (1) prescribed fires (Rx) or (2) drought-induced wildfires (Wild). Consequently, the combustion experiments included six permutations based on the ecoregion (P, CP, BR) and whether the burn was representative of a prescribed fire (Rx) or drought-induced wildfire (Wild): P-Rx, P-Wild, CP-Rx, CP-Wild, BR-Rx, and BR-Wild.

### 2.2 Preparation of Fuel Beds

We constructed fuel beds that replicated the average mass loadings of the different fuel components and 3D structures of fuel beds in each of the three ecoregions (P, CP, BR) based on extensive field sampling and light detection and ranging (LIDAR) measurements.^47–49^ The components of the surface fuels were categorized into fine and woody fuels, and the fine fuels were further separated into pine needles and other surface litter components. The woody fuels featured sticks of variable diameters and were categorized into 1, 10, and 100 h, which represent the time lags for the moisture content of the fuel to equilibrate with ambient relative humidity.^50^ The P and CP fuel beds featured surface fuels only, whereas the BR fuel beds also included a duff layer underneath the surface fuels.

The moisture content of the fuel beds was conditioned to represent either Wild or Rx conditions. Wildfires can occur at any moisture content, however, the majority of burned areas are consumed by wildfires that occur during drought conditions where the fuel beds are dry.^2,51^ Therefore, our Wild conditions represent drought-induced wildfires. The components of the Wild fuel beds were dried in an oven at 65 °C for 48 h leading to moisture content less than 4%, as quantified by a Moisture & Solids Analyzer (Computrac, Model MAX 4000XL), which are representative of dry fuel conditions as observed in the field.^52^ The fuel components of the Rx fuel beds were conditioned to moisture contents representative of prescribed fires in these regions.^2^ The fine fuels were first dried in the oven and then placed in a walk-in humidifier to bring their moisture content up to 10–11%. The woody fuels were first submerged in water until saturated, and then dried in the oven to a moisture content of 30–50%. The moisture content of the fine fuels is within the prescription window recommended by the U.S. Forest Service for southern ecosystems (8–15%),^53^ and the higher moisture content of the woody fuels is due to the longer time lag to adjust to atmospheric conditions, based on your experience in field observations of prescribed fires. In regions that contain duff, prescribed fires are recommended to be executed shortly after rainfall, such that the duff is too moist to be consumed.^21,54^ The duff had a moisture content of 50–60% when collected from the field, 2 days after rainfall, and was used as is in the BR-Rx fuel beds. The dry mass loadings and moisture contents for each fuel bed are given in Table 1.

**Table 1:** Mass Loading, Composition, and Moisture Content of the Fuel Beds^a^

ecoregion	dry mass loading	surface fuels composition	moisture content (wild)	moisture content (Rx)
Piedmont	surface: 0.5 kg	woody: 18%	woody: 1%	woody: 39%
(Mixed Forests)	duff: 0 kg	pine needles: 50%	fine: 2%	fine: 10.6%
		other litter: 32%		
Coastal Plain	surface: 0.5 kg	woody: 20%	woody: 0.8%	woody: 39%
(Woody Savanna)	duff: 0 kg	pine needles: 55%	fine: 2.4%	fine: 10.8%
		other litter: 24%		
Blue Ridge	surface: 0.2 kg	woody: 37%	woody: 1.7%	woody: 36%
(Deciduous Broadleaf Forest)	duff: 2.177 – 2.997 kg	litter: 63%	fine: 2.4%	fine: 10.4%
			duff: 2.6%	duff: 50–60%

a The designations in parentheses under each ecoregion represent broad mapping to land-cover types in the FINN emissions inventory.

The fuel beds created for these experiments had an area of 0.5 m^2^, which represents the scale of a “wildland fuel cell” unit, beyond which fire behavior becomes spatially independent.^55^ As such, these experiments aimed at capturing the combustion dynamics that contribute to smoke production.

### 2.3 Combustion Experiments

The combustion experiments were conducted in a 990 m^3^ burn room and the air inside the room was mixed using a fan to ensure well-mixed conditions of the smoke emissions. With the exception of BR-Wild, the burns typically concluded within 10 min, as inferred from real-time measurements of fuel consumption and fire radiative power (FRP), as elaborated in Sections 2.3.1 and 2.3.2. The BR-Wild burns, which involved duff ignition, lasted for significantly longer times (>60 min) due to prolonged duff smoldering. We note that even though the BR-Rx fuel bed also included duff, the high moisture content of duff in the BR-Rx burns (Table 1) rendered it unavailable for combustion. After conclusion of the burn, the smoke was sampled to an adjacent instrument room to perform various online measurements and collect filter samples for offline analyses.

#### 2.3.1 Fuel Consumption

The fuel beds were constructed on top of a scale (model # A&D GP-30KS) in order to monitor fuel consumption in real-time during the experiments. We calculated the dry fuel consumption (FC) as

2 $$\mathrm{FC}=\left(m_{1}-m_{2}\right)-\left(1-\mathrm{DC}\right)\left(m_{1}\right)$$

Where, DC is the fractional dry content of the fuel bed, and *m*_1_ and *m*_2_ are the fuel-bed masses measured by the scale preburn and postburn, respectively.

Equation 2 assumes that the residual mass in the fuel bed was completely dry.^44^ This is a reasonable assumption because we expect the vast majority of the moisture in the fuel bed to evaporate because of the heat generated by the combustion process. The only exception is BR-Rx. As noted earlier, the duff in the BR-Rx fuel bed was unavailable for combustion and postburn visual inspection indicated that it retained most of its moisture content. Therefore, DC in eq 2 was assumed to be that of the surface fuels. It is likely that a non-negligible amount of water evaporated from the duff in BR-Rx leading to overestimating the FC values.

#### 2.3.2 Fire Characterization Using Thermal Imagery

We monitored the fire behavior using two thermal imagers: FLIR A655 sc (herein referred to as FLIR) and Telops MS-M350 (herein referred to as Telops). FLIR has a broadband long-wavelength infrared channel (7.5–14 μm) and a resolution of 640 × 480 pixels, and was run in a temperature range of 100–2000 °C. Telops has a resolution of 640 × 512 pixels. It was customized with one broadband middle-wavelength infrared channel (1.5–5.4 μm) that can measure the background, and seven high dynamic range narrowband channels ranging between 1.6 and 4.7 μm that can measure fire temperatures of up to 1500 °C. Of primary importance for measuring FRP is the 3.98 μm channel that can measure between 250 and 1500 °C.

We converted the real-time combustion temperatures retrieved from FLIR and Telops into fuel-bed fire radiative power (FRP [W]) assuming gray-body radiation:^56^

3 $$\mathrm{FRP}=\sum \varepsilon •\sigma {•T}^{4}•A$$

Where, *T* is the temperature (K), ε is the emissivity (assumed to be 0.98),^56^ σ = 5.67 × 10^–8^ W m^–2^ K^–4^ is the Stefan–Boltzmann constant, and *A* is pixel area.

The FRP calculations in eq 3 were based on a minimum threshold of 300 °C,^57^ meaning that only pixels with temperatures above this threshold were used in the FRP calculations and that the burn was assumed to have concluded when the temperature in every pixel dropped below 300 °C. We also calculated the total fire radiative energy (FRE [MJ]) released by the burn by integrating FRP over the course of the burn. FRE, being the total radiative energy released from a fire, depends on the amount of fuel burned^41,42,58,59^ and is thus not indicative of combustion conditions. For instance, the same FRE could be obtained from a predominantly flaming fire with low fuel mass loading and a mostly smoldering fire with high mass fuel loading. Following Glenn et al.,^60^ we normalized FRE by the available fuel mass loading to obtain FRE_norm_ [MJ/kg]. FRE_norm_ can be thought of as an effective radiative heating value of the fuel bed and can be utilized as a proxy for combustion conditions.

### 2.4 Quantifying Elemental Carbon (EC) and Organic Carbon (OC) Emissions

We calculated the mass of EC and OC emitted by each burn as

4 M=C×∀

Where, ∀ = 990 m^3^ is the volume of the burn room and *C* is the mass concentration of EC or OC in the burn room, which was calculated as

5 $$C=\frac{m}{\mathrm{Qt}}K$$

Where, *m* is the mass loading of EC or OC on the filter obtained using thermal-optical analysis, Q is the flow rate through the filter (5 SLPM), t is the filter collection time (15–35 min), and K is a correction factor that accounts for particle losses in the burn room (see SI for details).

The thermal-optical analysis to quantify OC and EC mass loadings on the filter samples followed the same procedure described in our previous studies.^60–62^ We collected quartz (Q) and quartz behind Teflon (QBT) filters and analyzed them using the Niosh-870 ^63^ protocol in an OCEC analyzer (Sunset Laboratory, Model 5 L). EC mass loading was determined from the Q filters, whereas the OC mass loading was corrected for vapor adsorption by subtracting the OC mass loading on the QBT filters from the OC mass loading on the Q filters.^64^

### 2.5 Quantifying Secondary Organic Carbon (SOC) Formation

The smoke emissions were oxidized with OH radicals in an oxidation flow reactor (OFR) (Aerodyne).^65–67^ The OFR consists of a 13 L cylindrical flow equipped with low-pressure mercury lamps that emit UV light at wavelengths of 185 and 254 nm. In this study, the OFR was operated in the OFR185 mode, which produces OH radicals from the following reactions:^68^

$$H_{2}O+\mathrm{hv}\left(185\mathrm{nm}\right)\to \mathrm{OH}+H$$

$$O_{2}+\mathrm{hv}\left(185\mathrm{nm}\right)\to O{(}^{3}P)+O{(}^{3}P)$$

$$O{(}^{3}P)+O_{2}\to O_{3}$$

$$O_{3}+\mathrm{hv}\left(254\mathrm{nm}\right)\to O{(}^{1}D)+O_{2}$$

$$O{(}^{1}D)+H_{2}O\to 2\mathrm{OH}$$

OH exposure in the OFR is a function of residence time, UV light intensity, water vapor concentration, temperature, and OH reactivity (the rate at which OH is scavenged by smoke constituents). The flow rate through the OFR was 10 SLPM, resulting in a constant average residence time of 1.3 min throughout the experiments. The UV light intensity was also kept constant at a voltage setting of 2.2 V throughout the experiments. The water vapor concentration, however, was not controlled in the experiments and was dictated by the ambient conditions for each day (Table S1). Similarly, the temperature in the OFR exhibited day-to-day variability (Table S1). Furthermore, though not quantified in our experiments, OH reactivity is expected to exhibit some variation across experiments. Table S1 lists OH exposure for each experiment as estimated by the OFR software based on the parametrization of Li et al.^68^ We note that these OH exposure estimates do not account for OH reactivity and should be considered as upper limits.

We estimated organic aerosol (OA) enhancement^33,36,38^ from the difference in integrated SMPS aerosol concentrations between the fresh aerosol (with OFR lights turned off) and aged aerosol (with OFR lights turned on), as illustrated in Figure S1. We assumed that the OA enhancement was equivalent to SOA formation. Previous studies have reported that the depletion of some semivolatile organic compounds (SVOCs) due to oxidation can lead to partitioning of these SVOCs from the particle phase to the gas phase, thus reducing the OA concentration.^69^ Therefore, it is possible that the SOA formation was larger than estimated from OA enhancement, though we do not expect this effect to be significant. The amount of SOA formation also depends on the mass of existing OA, which affects the extent of gas-particle equilibrium partitioning,^69^ as well as the aerosol condensation sink, which dictates the partitioning (condensation) time scales.^70,71^ The amount of aerosol production was highly variable across experimental permutations (Section 3.3). In order to minimize the effect of variability in aerosol concentration on SOA formation, we vented a fraction of the smoke in the burn room before starting the OFR measurements to bring the aerosol volume concentrations and condensation sinks to levels that were consistent across experiments within approximately a factor of 2 (Table S1).

To bring SOA formation to common grounds that can be compared with OC and EC emissions, we calculated secondary organic carbon (SOC) emissions as the product of OA enhancement with OC emissions.

## 3 Results and Discussion

### 3.1 Fire Radiative Power and Fire Radiative Energy

Figure 1 depicts representative FRP time series retrieved from FLIR and Telops for each of the six experimental permutations. The two cameras show similar fire behavior, including start and end of the burn, location of peak-FRP associated with the flaming phase of the burns, the prolonged low-intensity smoldering in the BR-Wild burn, as well as occasional flare-ups that occurred after the major flaming phase, which manifest as small peaks in FRP. However, the FRP retrieved from Telops was consistently larger than FLIR.

**Figure 1:**
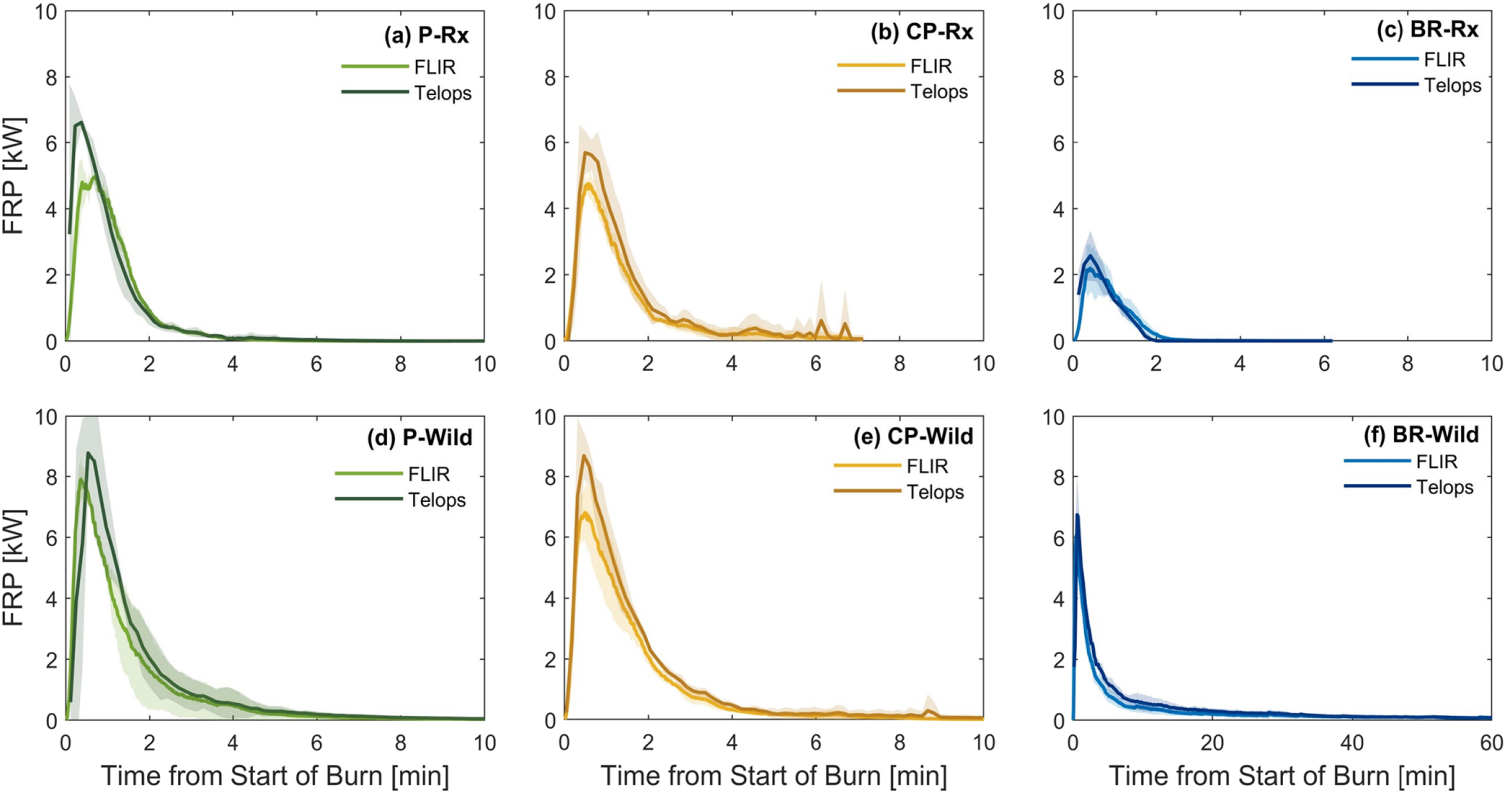
Time series of fire radiative power (FRP) from each experimental permutation obtained from FLIR and Telops. Solid lines are averages and shaded areas are standard deviations. (a) P-Rx (10/28/2022), (b) CP-Rx (11/07/2022), (c) BR-Rx (11/10/2022), (d) P-Wild (10/27/2022), (e) CP-Wild (11/02/2022), and (f) BR-Wild (11/09/2022). Note difference in in *x*-axis scale between BR-Wild (f) and the rest of the panels.

Despite the difference in magnitude, FRP retrieved from the two cameras exhibit the same trends across experimental permutations. For P and CP, which included surface fuels only and had the same dry mass loading (Table 1), the higher moisture content in Rx led to a lower peak-FRP compared to Wild due to the moisture consuming part of the energy released from the combustion process for vaporization, which also led to overall suppression of fuel consumption (Section 3.2). Despite the high mass loading of duff in BR, the duff did not ignite in BR-Rx and the FRP was released solely from combustion of the surface fuels. The lower peak-FRP in BR-Rx compared to P-Rx and CP-Rx is due to the lower mass loading of surface fuels in BR-Rx (Table 1). Duff ignited in BR-Wild but had minimal contribution to the high-temperature phase of the combustion as evidenced by the peak-FRP in BR-Wild being lower than P-Wild and CP-Wild. However, the duff exhibited prolonged combustion at low temperatures (smoldering) as evidenced by the long FRP tail. The implications of the substantially different combustion regime in BR-Wild compared to the other five experimental permutations that did not involve duff combustion are further elaborated in the subsequent sections.

The trends in FRP described in the previous paragraph are more compactly visualized via the FRE values obtained from all experiments, shown in Figure 2. BR-Rx had the smallest FRE because of the low mass loading of surface fuels and because duff did not ignite. For P and CP, the higher moisture content in Rx led to a sizable reduction in FRE compared to Wild. Despite its relatively low peak-FRP, BR-Wild had the largest FRE due to the high mass loading of duff, which smoldered at low FRP for significantly longer times compared to the other experimental permutations.

**Figure 2:**
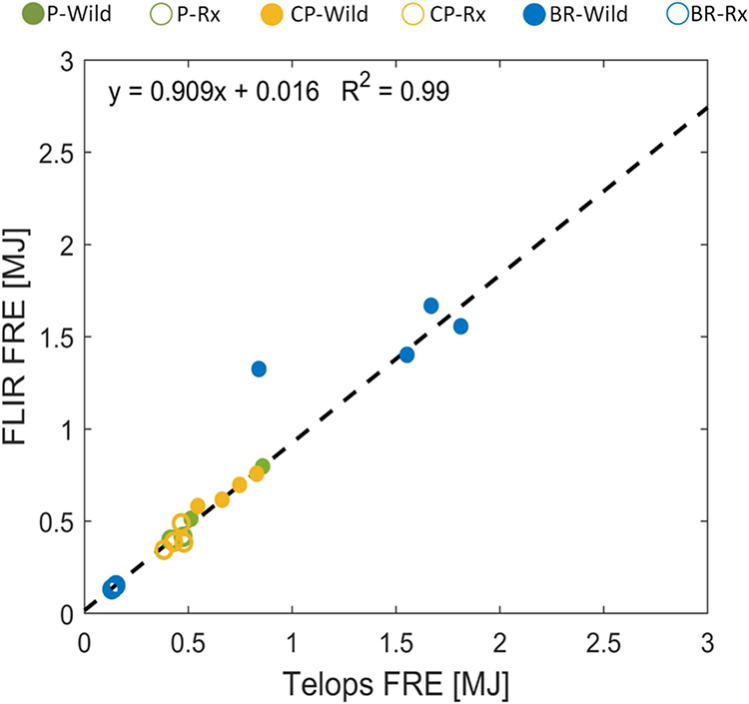
Fire radiative energy (FRE) obtained from FLIR and Telops. The linear fit excludes the outlier BR-Wild data point. Numerical values are listed in SI Table S2.

With the exception of one outlier BR-Wild experiment, the FRE values obtained from the two cameras are well-correlated with a slope close to unity and a small intercept. The difference in FRE retrieved from the two cameras did not have significant implications on the major findings of this study regarding the dependence of smoke emissions on combustion conditions, as elaborated in the subsequent sections.

### 3.2 Fire Radiative Energy Correlated with Fuel Consumption

Figure 3 depicts FRE versus fuel consumed for each experiment. As expected, an increase in FRE is associated with an increase in fuel consumption because FRE represents the total amount of radiative energy release from a burn and is largely dependent on the amount of fuel burned. Furthermore, the reasons behind the differences in FRE between the experimental permutations that are discussed in Section 3.1 also apply to fuel consumption. For P and CP, the higher moisture content in Rx led to consistently less fuel consumption compared to Wild. Similar dependence of litter fuel consumption on moisture content was reported for field observations of Southeastern prescribed fires.^21^ The large difference in fuel consumption between BR-Wild and BR-Rx is because duff underwent ignition in Wild but was unavailable for combustion in Rx, which is consistent with field reports that the presence of duff leads to large differences in fuel consumption between wildfires and prescribed fires.^20^

**Figure 3:**
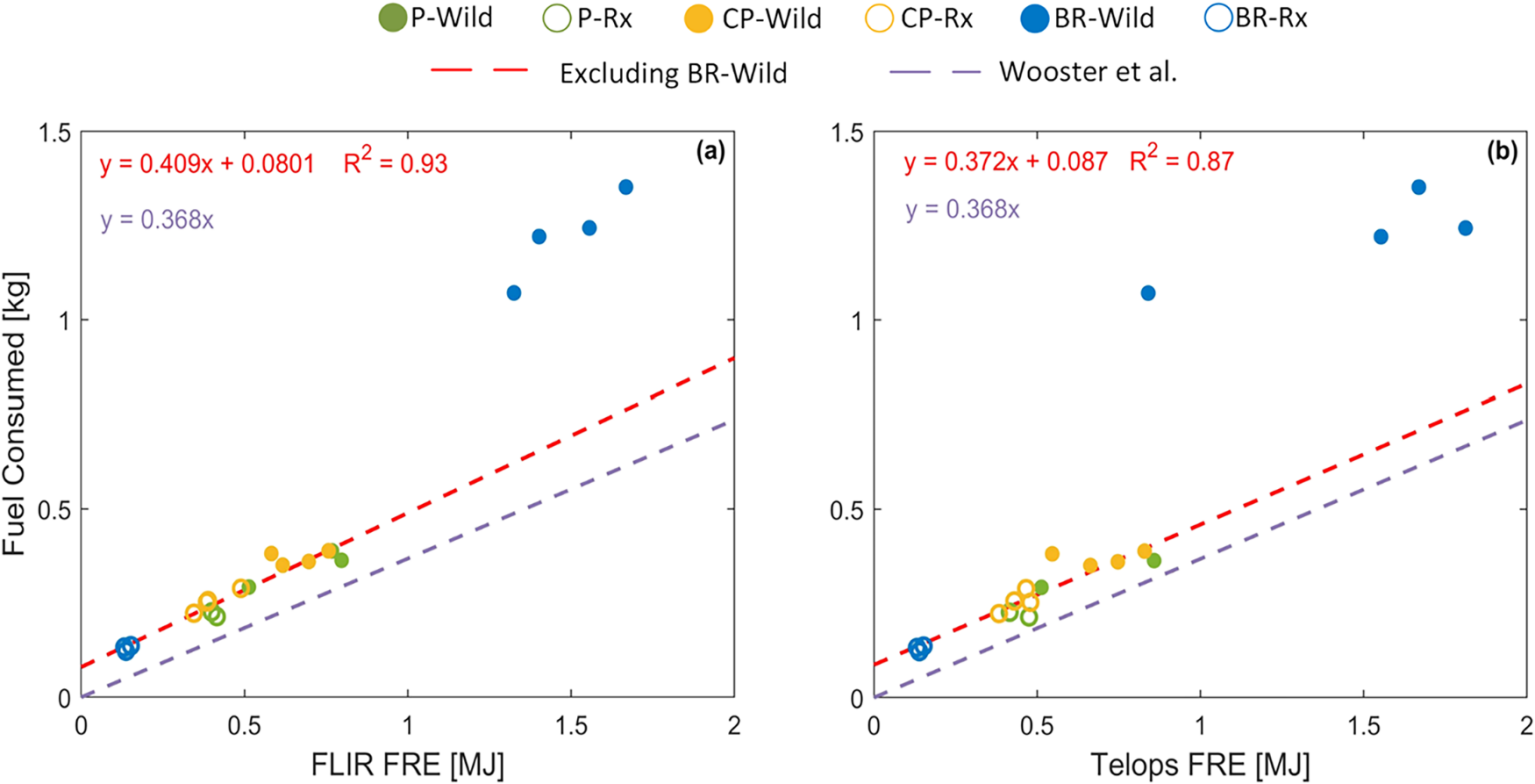
Fuel consumed versus fire radiative energy (FRE) obtained from (a) FLIR and (b) Telops. The red dashed line is a linear fit to data points from experimental permutations that did not involve duff combustion (i.e., excluding BR-Wild). The offset in the fit (positive fuel consumption at FRE = 0) is likely due to the threshold of 300 °C used in the FRP calculations (Section 2.3.2). The purple dashed line is a linear fit reported by Wooster et al.^41^ Numerical values are listed in SI Table S3.

As shown in Figure 3, FRE and fuel consumption are well-correlated for the experimental permutations that did not involve duff combustion (i.e., excluding BR-Wild). Also shown is a linear fit reported by Wooster et al.^41^ for burns that involved various herbaceous and woody fuels. Bearing in mind the differences in experimental setups between the two studies, the Wooster et al. fit approximates our data relatively well, with the exception of the BR-Wild data points, which exhibit substantially larger amounts of fuel burned per unit FRE. This finding points to differences in combustion conditions between BR-Wild and the other experimental permutations, which did not involve duff combustion. Specifically, duff combusts less efficiently than surface fuels and thus releases less radiative energy per unit mass fuel burned. A practical implication of these results is that an assumed linear relationship between FRE and fuel consumption is useful for predicting fuel consumption from FRE measurements for wildland fires that consume surface fuels. However, applying this relationship to ground fires that also consume duff would lead to underestimation of duff consumption.

### 3.3 Elemental Carbon (EC) Emissions, Organic Carbon (OC) Emissions, and Secondary Organic Carbon (SOC) Formation Correlated with Fire Radiative Energy

Ichoku et al. reported that particulate matter (PM) emissions from the combustion of various fine and small woody (1 h) biomass fuels were linearly correlated with FRE^42^ and demonstrated the utility of this correlation in building a top-down emission inventory based on satellite observations of FRP and aerosol optical depth.^72^ Here, we investigate the applicability of this framework to speciated carbonaceous aerosol emissions, namely EC and OC, as well as SOC formation. As shown in Figure 4, the P and CP fuel beds (P-Wild, P-Rx, CP-Wild, and CP-Rx), which had the same mass loading, had similar total OC emissions. The reason is that P-Wild and CP-Wild had smaller OC emission factors (g/kg-fuel burned) but larger amounts of fuel burned (available fuel loading × fuel consumption fraction) compared to P-Rx and CP-Rx (Table 2), leading to similar amounts of total OC emissions (g). As expected, BR-Rx had the lowest OC emissions because the BR fuel bed had the smallest mass loading of surface fuels and duff was not available for combustion, and BR-Wild had the largest OC emissions because duff was available for combustion. Despite the influence of moisture content on OC emission factors (Section 3.5) and on FRE (Sections 3.1 and 3.2), fuel mass loading and availability were more important in dictating the overall trends in OC emissions and FRE. This is manifested in a good linear correlation between OC emissions and FRE with a relatively small intercept (Figure 4c,d), suggesting that FRE can be used as a first-order predictor of OC emissions.

**Table 2:** Available Fuel Loading, Fuel Consumption Fraction, EC and OC Emission Factors, and SOC Formation Factors for Each Experimental Permutation^a^

experimental permutation	available fuel loading [kg]	fuel consumption fraction [%]	EC emission factor		OC emission factor		SOC formation factor	
			g/kg	g/m^2^	g/kg	g/m^2^	g/kg	g/m^2^
P-Wild	0.501 (0.00082)	69.5 (10.01)	0.6 (0.11)	0.40 (0.117)	7.4 (1.145)	5.14 (0.0477)	2.33 (0.501)	1.61 (0.222)
P-Rx	0.496 (0.00099)	44.34 (1.697)	0.412 (0.0174)	0.22 (0.0052)	13.34 (2.071)	6.99 (0.900)	3.78 (1.723)	2.00 (0.960)
CP-Wild	0.503 (0.00352)	73.5 (3.74)	0.687 (0.192)	0.51 (0.125)	6.24 (0.84)	4.67 (0.834)	1.66 (0.767)	1.24 (0.636)
CP-Rx	0.495 (0.0023)	51.5 (5.33)	0.452 (0.0797)	0.27 (0.0661)	11.01 (1.94)	6.50 (1.434)	4.90 (0.349)	2.80 (0.341)
BR-Wild	2.96 (0.254)	41.4 (2.99)	0.117 (0.029)	0.29 (0.0685)	6.89 (2.39)	17.70 (6.771)	8.36 (2.906)	21.76 (6.621)
BR-Rx	0.200 (0.000)^b^	65.3 (3.74)	0.537 (0.069)	0.14 (0.0229)	9.59 (1.68)	2.49 (0.346)	4.29 (1.824)	1.12 (0.495)

a Data are presented as average (standard deviation). Emission and formation factors are presented per unit mass of fuel burned and per unit area of fuel bed.

b Only surface fuels are included because duff was not available for combustion in BR-Rx.

**Figure 4:**
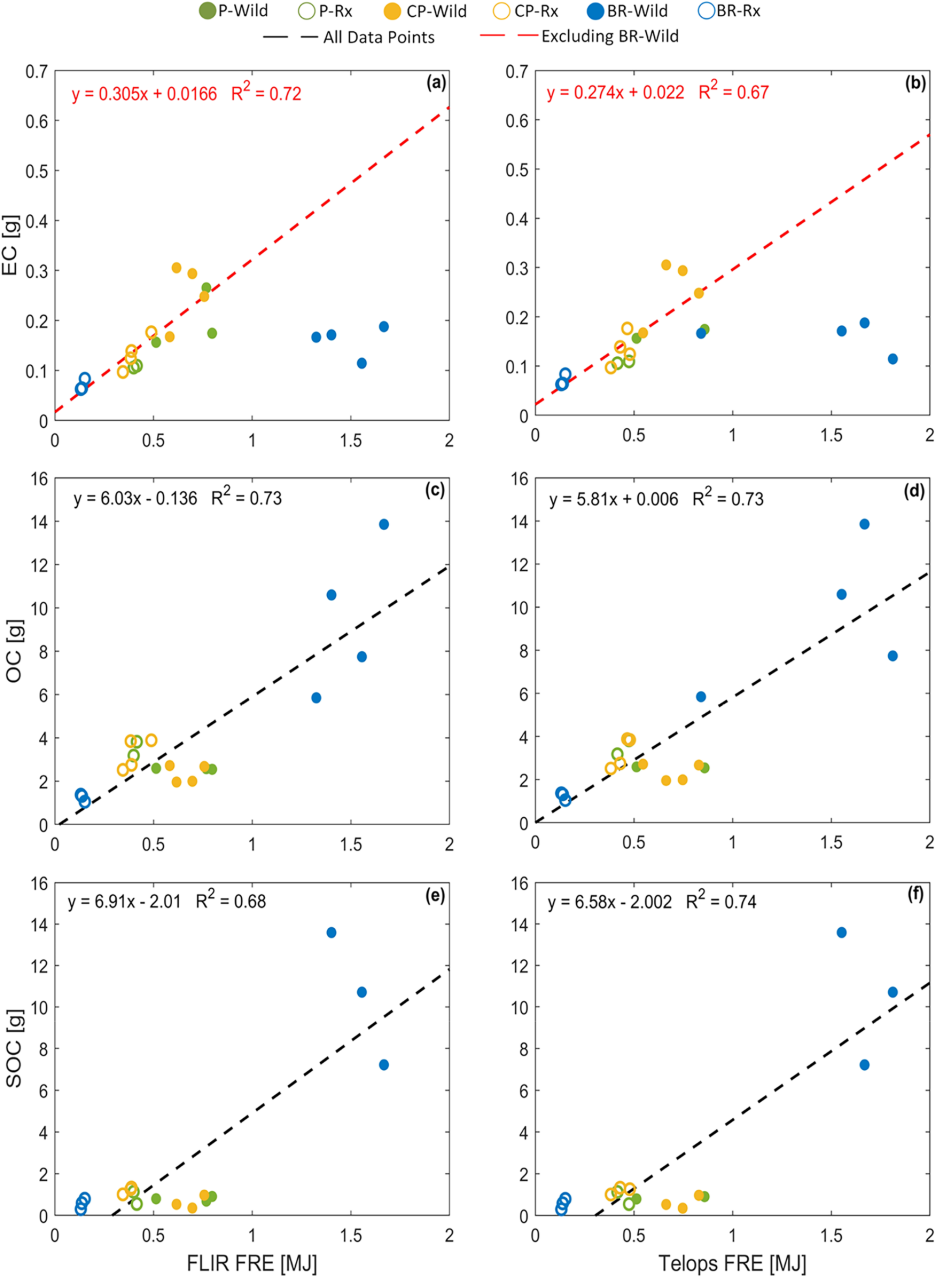
Emissions of (a, b) elemental carbon (EC) and (c, d) organic carbon (OC), and (e and f) formation of secondary organic carbon (SOC) versus fire radiative energy (FRE). The left panels (a, c, e) correspond to FRE obtained from FLIR and the right panels (b, d, f) correspond to FRE obtained from Telops. The black dashed lines are linear fits to data points from all experimental permutations and the red dashed lines are linear fits to data points from experimental permutations that did not involve duff combustion (i.e., excluding BR-Wild). Numerical values are listed in SI Table S4.

The picture, however, is different for EC emissions. For experimental permutations that involved combustion of surface fuels only (excluding BR-Wild), FRE is better correlated with EC emissions than OC emissions. This is due to the higher FRE in P-Wild and CP-Wild compared to P-Rx and CP-Rx, which is associated with higher peak-FRP (Figure 1) and is indicative of more flaming combustion that is conducive for EC formation. However, EC emissions in BR-Wild fall significantly below the correlation obtained from the experiments that involved combustion of surface fuels only. The reason is that the high FRE in BR-Wild is due to the prolonged combustion at low FRP (Figure 1), which is indicative of smoldering combustion that is not conducive for EC formation.

These results suggest that FRE can be a practical metric for predicting total OC emissions from a burn. This approach is appealing because it does not require knowledge of fuel loading, fuel availability, combustion completeness, emissions factors, and how they depend on environmental conditions (e.g., moisture content). Put in other words, the multidimensional problem of predicting OC emissions from the fuel-bed variables listed above can be effectively reduced to a single dimension, namely FRE. However, whereas this approach can also be applied to EC for situations that do not involve duff ignition, it would severely overestimate EC emissions when duff is available for combustion. As further elaborated in Section 3.4, predicting EC emissions requires knowledge of fuel availability and combustion conditions. We note that OC emissions are 1–2 orders of magnitude larger than EC (Figure 4) and primary organic aerosol (POA) – i.e., OC and other noncarbon constituents of the particulate organics – constitutes the majority of PM in wildland-fire smoke. Therefore, despite the discrepancy in EC emissions associated with duff combustion, our results are in agreement with the report of Ichoku et al.^42^ that FRE is a good metric for predicting PM emissions. We should also note that quantitative translation of FRE-based parametrizations from laboratory experiments or surface field measurements to air-craft or remote-sensing observations is not straightforward and is a subject of ongoing investigation. Specifically, airborne and satellite FRE measurements can be biased due to integration of areas not burning, canopy obscuration, missing small fires (especially prescribed fires), among other issues.

BR-Wild, which had significantly higher FRE compared to the other experimental permutations, also had significantly higher levels of SOC formation (Figure 4e,f). However, the linear correlation between SOC formation and FRE had a relatively large (negative) intercept, indicating that FRE is not a good predictor of SOC formation. This is attributed to the dependence of SOC formation potential on combustion conditions, as elaborated in Section 3.4.

### 3.4 Combustion Conditions

We invoked variability in combustion conditions in the previous sections to explain differences in EC and OC emissions and SOC formation between the different experimental permutations in this study. Here, we present a more focused analysis of the effect of combustion conditions. To help set the stage for this discussion, it is beneficial to start with the simple conceptual model shown in Figure 5. Going from left to right in combustion conditions space (*x*-axis in Figure 5) is associated with more complete conversion of carbon in the fuel to CO_2_, which is associated with more heat release from the combustion process,^73^ thus higher combustion temperatures. In the combustion of simple hydrocarbon fuels, EC, OC, and SOC precursors (i.e., organic species in the vapor phase) are incomplete-combustion (intermediate) products with temperature-dependent emission profiles. The formation of these species is more complex in the combustion of biomass fuels. The heat release from the combustion process induces reactions in the biomass matrix, such as depolymerization, aromatization, fragmentation, and distillation that lead to the emission of various complex organic species.^74^ There is evidence that the majority of volatile organic compounds in wildland fires are not products of combustion but rather products of biomass pyrolysis and distillation induced by the combustion process.^75–78^ Nevertheless, the overlapping combustion, pyrolysis, and distillation processes are still expected to result in temperature-dependent (i.e., combustion-condition-dependent) emission profiles of EC, OC, and SOC precursors. Starting from the left end of the combustion conditions space, the low temperatures encountered in these conditions are conducive for the emission of small organic species that would exist solely in the vapor phase at ambient conditions due to their high volatility. These species can be SOC precursors. Further increase in combustion temperatures makes the formation of larger organic species (such as polycyclic aromatic hydrocarbons) more thermodynamically favorable.^79^ These large organic species have low volatilities and thus partition to the particle phase and form organic aerosol (i.e., OC). Further increase in combustion temperature leads to conditions that are more conducive for the completion of the soot-formation process^80^ and are thus marked by increase in EC emissions. The emissions of all the intermediate carbonaceous species eventually drop with increasing temperature, as the conversion of carbon in the fuel to CO_2_ becomes more efficient in the major combustion process. Furthermore, any organics released from the biomass matrix would also reduce to CO_2_ (i.e., combust) at high temperatures. In the context of biomass combustion, this high-efficiency combustion could be actualized by say grinding the biomass into small particles and performing the combustion in a fluidized bed to enhance fuel-air mixing. Overall, we expect the emission profiles of all carbonaceous species to peak at certain combustion conditions (temperatures), with the temperatures corresponding to peak emissions being the highest for EC, followed by OC and SOC precursors.

**Figure 5:**
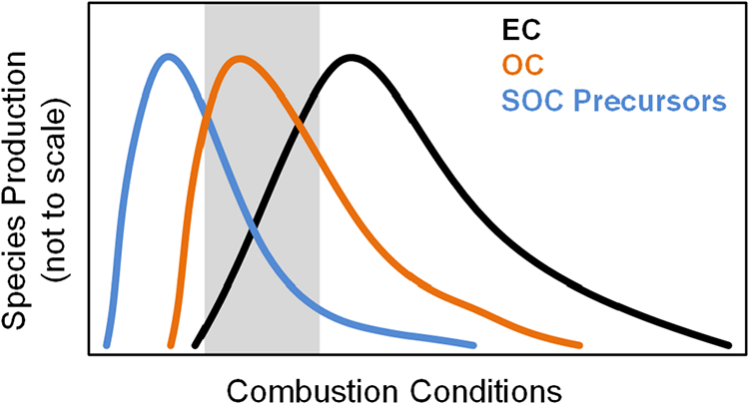
Conceptual model of the production of elemental carbon (EC), organic carbon (OC), and secondary organic carbon (SOC) precursors as a function of combustion conditions. Going from left to right in combustion conditions space is associated with greater release of radiative energy, thus higher combustion temperatures. The gray shaded area represents the likely range of combustion conditions encountered in our experiments.

As described in Section 2.3.2, we use FRE normalized by dry mass loading of available fuel (FRE_norm_ [MJ/kg]) as a proxy for combustion conditions.^60^ The effects of combustion conditions (via FRE_norm_) on the emission factors of EC (EF_EC_) and OC (EF_OC_) in g per kg fuel burned are illustrated in Figure 6. To bring SOC formation to the same basis as EF_EC_ and EF_OC_, we define SOC formation factor (FF_SOC_) as the formation potential of SOC in g per kg fuel burned. EF_EC_ increases with increasing FRE_norm_, FF_SOC_ decreases with increasing FRE_norm_, and EF_OC_ increases, peaks at ∼0.8 MJ/kg, then decreases with increasing FRE_norm_. Following from the conceptual model in Figure 5, these results suggest that our experiments encompass a range of combustion conditions that capture the right tail in production of SOC precursors, the left tail of EC production, and the peak of OC production.

**Figure 6:**
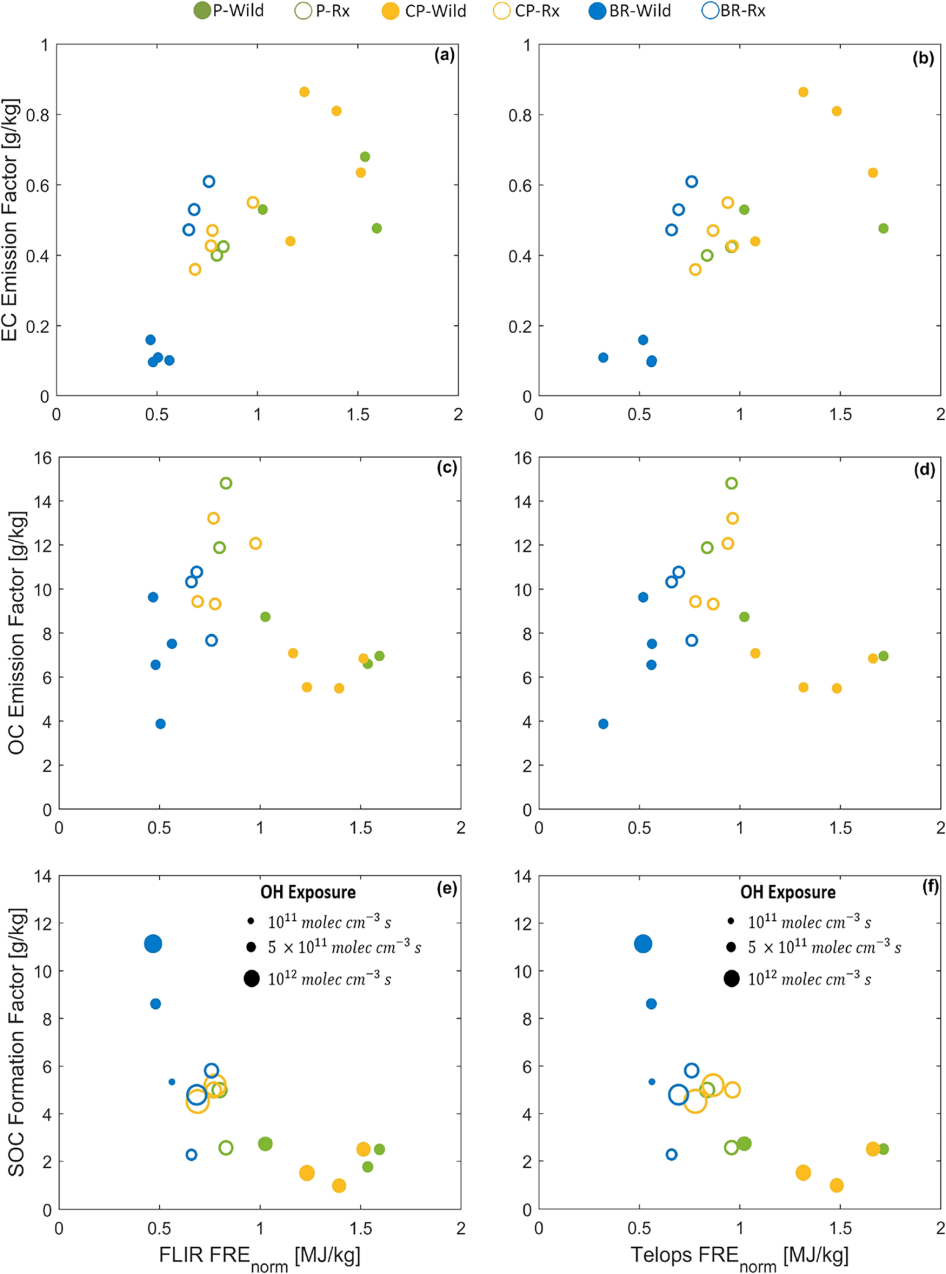
(a, b) Elemental carbon emission factors (EF_EC_), (c, d) organic carbon emission factors (EF_OC_), and (e, f) secondary organic carbon formation factors (FF_SOC_) versus fire radiative energy normalized by fuel-bed mass loading (FRE_norm_). The left panels (a, c, e) correspond to FRE_norm_ obtained from FLIR and the right panels (b, d, f) correspond to FRE_norm_ obtained from Telops. Numerical values are listed in SI Table S5.

EC is produced via the soot-formation route associated with the combustion process, which supports the validity of the dependence of EF_EC_ profile on combustion conditions (i.e., FRE_norm_) across all experimental permutations. The dependence of EF_OC_ and FF_SOC_ on FRE_norm_ across all experimental permutations, however, requires further validation. For P and CP, the reduction in both EF_OC_ and FF_SOC_ with increasing FRE_norm_ can be attributed to differences in combustion conditions because of the similarity in fuel-bed composition. However, it is possible that the EF_OC_ and FF_SOC_ trends associated with BR-Wild are not entirely governed by combustion conditions but are in part a consequence of fuel-bed composition, specifically the existence of duff. Nevertheless, the results shown in Figure 6 provide evidence that combustion conditions are, at least in part, implicated in the large variability in biomass-burning SOC formation reported in different studies.^14,19^

As described in Section 2.5, there are several parameters (other than combustion conditions) that varied across the different experiments and could potentially affect the extent of SOC formation. Those include OH exposure, OFR temperature, as well as aerosol mass concentration and condensation sink. As described in Section 2.5, OH exposure exhibited variability across experiments. As illustrated in Figure 6e,f, even though OH exposure can explain some of the variability in FF_SOC_ for each experimental permutation (mostly BR-Wild), differences in combustion conditions (FRE_norm_) across permutations exhibit a stronger effect on FF_SOC_. For completeness, Figure S2 shows FF_SOC_ as a function of OH exposure. Despite variability over an order of magnitude, OH exposure does not impose a trend on SOC formation. Similarly, variabilities in OFR temperature (Figure S3), aerosol volume concentration (Figure S4), and aerosol condensation sink (Figure S5) do not impose a trend on SOC formation. The results in Figures S2–S5 indicate that despite the uncertainty associated with variability of these parameters across experiments, the trends in Figure 6e,f support our framework regarding the dependence of SOC formation on combustion conditions (Figure 5).

One practical application that emerges from the dependence of EC and OC emissions on FRE_norm_ is utilizing this dependence to quantify EC emissions from wildland fires that involve combustion of surface fuels and duff. As described in Section 3.3 and shown in Figure 4, OC emissions can be predicted from FRE for all experimental permutations but EC emissions from BR-Wild, which involved duff combustion, fell below the correlation obtained from experimental permutation that involved combustion of surface fuels only. As shown in Figure 7, EC/OC is linearly correlated with FRE_norm_ for all experimental permutations. Therefore, observations of FRE^72,81,82^ can be used in conjunction with observations of fuel loading^83–87^ to obtain both OC and EC emissions.

**Figure 7:**
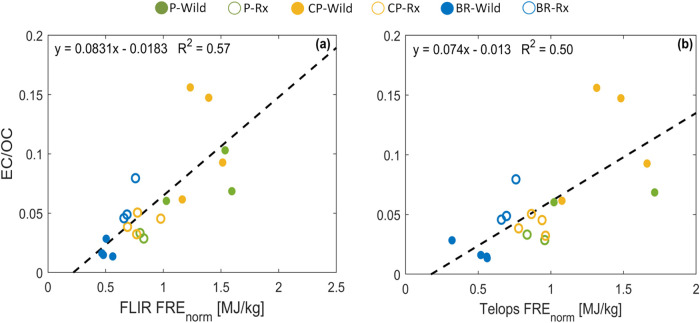
Ratio of elemental carbon (EC) emissions to organic carbon (OC) emissions versus fire radiative energy normalized by fuel-bed mass loading (FRE_norm_) calculated using (a) FLIR and (b) Telops. Numerical values are listed in SI Table S6.

### 3.5 Wildfires versus Prescribed Fires

Here, we discuss the effect of differences in combustion conditions, which arise from differences in fuel-bed composition and moisture content, on fuel consumption, emission factors of EC and OC, and formation factors of SOC within the context of prescribed fires and wildfires.

Table 2 presents fuel consumption fractions (FCF), emission factors of EC (EF_EC_) and OC (EF_OC_), and formation factors of SOC (FF_SOC_) for each experimental permutation. Focusing first on the P and CP fuel beds, which included surface fuels only, the results suggest a weak influence of the variability in fuel bed composition between the two ecoregions on FCF and EF. Averaged over all Rx and Wild burns, P had a slightly smaller FCF compared to CP (59.4 vs 62.5%), slightly smaller EF_EC_ (0.50 vs 0.57 g/kg), slightly larger EF_OC_ (9.80 vs 8.62 g/kg), and slightly smaller FF_SOC_ (3.06 vs 3.28 g/kg). These trends, though weak, can be attributed to differences in fuel-bed composition. The CP fuel bed contained appreciable amounts of grass litter and no oak leaves, whereas the P fuel bed contained appreciable amounts of oak leaves and no grass litter.^60,88^ It is likely that the grass litter in the CP fuel bed combusted more completely and efficiently than the leaves in the P fuel bed, resulting in the trends in EF_EC_ and EF_OC_ listed above. We note that FF_SOC_ is also governed by OH exposure, which was on average higher in the CP experiments compared to the P experiments (Tables S1), and could explain the slightly larger FF_SOC_ in the CP experiments. When averaged over both P and CP fuel beds, Rx had smaller FCF compared to Wild (47.9 vs 71.5%) smaller EF_EC_ (0.43 vs 0.64 g/kg), larger EF_OC_ (12.18 vs 6.82 g/kg), and larger FF_SOC_ (4.34 vs 1.99 g/kg). These results indicate that the influence of moisture content was more prominent than fuel-bed composition in modulating FCF, EF_EC_, EF_OC_, and FF_SOC_. Furthermore, the results convey a clear trend where lower moisture content leads to more combustion completeness (higher FCF), which is associated with higher production of EC and lower production of OC and SOC precursors. The dependence of FCF on moisture content in P and CP fuel beds is consistent with field measurements of litter consumption in Southeastern prescribed fires.^21^

BR-Rx had higher average FCF (65.30%) compared to P-Rx (44.34%) and CP-Rx (51.50%). This could be due to the lower mass loading of surface fuels in the BR fuel bed (0.2 kg) compared to P and CP (0.5 kg), which could lead to more efficient flame propagation. However, we expect that this relatively high FCF is partly due to overestimating fuel consumption in BR-Rx (Section 2.3.1). Nevertheless, the average FCF in all Rx experiments in this study (54.5%) is close to the average FCF reported by Reid et al.^89^ (52%) in prescribed fires in the Southeastern U.S. (Northern Florida and Southern Georgia). Conversely, BR-Wild had substantially lower average FCF (41.4%) compared to P-Wild (69.5%) and CP-Wild (73.5%). The reason is that duff was available for combustion in BR-Wild. Despite having low moisture content in BR-Wild (<3%), the higher bulk density of duff compared to surface fuels^90^ leads to more oxygen-deprived combustion,^20,91,92^ thus suppressing combustion completeness. The low-temperature oxygen-deprived combustion regime associated with duff was manifested in substantially lower EF_EC_ in BR-Wild (0.12 g/kg) compared to P-Wild (0.60 g/kg) and CP-Wild (0.69 g/kg). This inefficient combustion also led to substantially higher production of SOC precursors, thus higher FF_SOC_ in BR-Wild (8.36 g/kg) compared to P-Wild (2.33 g/kg) and CP-Wild (1.66 g/kg). EF_OC_ in BR-Wild (6.89 g/kg) was bounded by CP-Wild (6.24 g/kg) and P-Wild (7.4 g/kg). However, we attribute this similarity in EF_OC_ to BR-Wild on one hand and P-Wild and CP-Wild on the other hand being on opposite ends of OC-production profiles *vis-à-vis* combustion conditions, as discussed in Section 3.4

Overall, our results highlight the importance of moisture content in modulating fuel availability and consumption, combustion conditions, and consequently EF_OC_, EF_EC_, and FF_SOC_. Fuel availability is key, especially for fuel beds that contain duff, which typically has substantially larger mass loadings compared to surface fuels (Table 1). Therefore, if duff is available for combustion, as is the case during drought conditions, fuel consumption can be an order of magnitude larger than the case where duff is not available for combustion.^25^ Consequently, for the same fuel bed that contains duff, OC emissions and SOC formation per unit area can be an order of magnitude larger for a drought-induced wildfire, where duff is available for combustion, compared to a prescribed fire, where duff combustion is purposefully avoided (Table 2).^20^

It is important to note that duff formation depends on the recent fire history. By removing fine surface fuels, continuous prescribed burning helps arrest duff formation. Furthermore, in regions where duff had already formed, duff ignition is typically predicated on the ignition of fine surface fuels. Therefore, the consumption of fine surface fuels in prescribed fires in regions that contain duff, though emits smoke, can potentially prevent significantly higher levels of smoke production that would occur in a wildfire that consumes both surface fuels and duff. It is also important to note that the small energy release associated with duff combustion leads to low injection heights of the emitted smoke. Furthermore, the elongated duff burning often continues through the night. The stable atmospheric conditions at night coupled with the low injection heights result in minimal vertical mixing of the smoke emissions and consequently, high surface-level smoke concentrations. This highlights the utility of prescribed fires from the perspective of reducing the likelihood of severe smoke episodes associated with duff combustion.

## Supplementary Material



## Notes

Correction for wall losses, figures for corrections and OA enhancement, and tables which provide data for OH exposure, fuel consumption, FRE (both cameras), emissions of EC and OC, and formation of SOC (PDF)

## #

Aerodyne Research Inc., Billerica, Massachusetts 01821, USA

## ∇

Tofwerk USA, Boulder, Colorado 80301, USA.
