# Supplementary material for: The Effect of Combustion Conditions on Emissions of Elemental Carbon and Organic Carbon and Formation of Secondary Organic Carbon in Simulated Wildland Fires
Source: ACS EST Air. 2025 Sep 11;2(10):2056–70. doi: 10.1021/acsestair.4c00300 (PMC12519481; doi:10.1021/acsestair.4c00300)
Supplement: Supplementary file 1 [file ea4c00300_si_001.pdf]

Supporting Information for:

The Effect of Combustion Conditions on Emissions of Elemental Carbon and Organic Carbon and Formation of Secondary Organic Carbon in Simulated Wildland Fires

Robert Penland [a], Steven Flanagan [b], Luke Ellison [c], Muhammad Abdurrahman [a], Chase K. Glenn [a‡], Omar El Hajj [a†], Anita Anosike [a], Kruthika Kumar [a], Mac A. Callaham [b], E. Louise Loudermilk [b], Nakul N. Karle [d], Ricardo K. Sakai [d], Adrian Flores [d], Tilak Hewagama [e], Charles Ichoku [c], Joseph O'Brien [b], Rawad Saleh [a]*

[a] *School of Environmental, Civil, Agricultural, and Mechanical Engineering, University of Georgia, Athens, GA, USA*

[b] *USDA Forest Service Southern Research Station, Athens, Georgia, USA*

[c] *University of Maryland – Baltimore County, Baltimore, Maryland, USA*

[d] *Howard University Beltsville Campus, Beltsville, Maryland, USA*

[e] *NASA Goddard Space Flight Center, Greenbelt, Maryland, USA*

[‡] *Now at Aerodyne Research Inc., Billerica, Massachusetts, USA*

[†] *Now at Tofwerk USA, Boulder, Colorado, USA*

S1. Correction for Particle Losses in the Burn Room

Particle concentrations in the burn room exhibited a decay due to infiltration and wall-losses. To account for these losses, we performed box model calculations that account for particle emissions and losses:

$$\frac{dC}{dt} = \dot{E} + \frac{Q}{\forall}(C^b - C) \qquad \text{(S1)}$$

Where:

C is the particle mass concentration in the burn room;

C^b is the ambient (background) particle mass concentration of the species outside the burn room, which was much smaller than the particle concentration in the burn room and can be neglected;

$\dot{E}$ is the time-dependent emission rate of the particles from the burn per unit volume of air in the burn room, which has units of mass concentration per time (e.g., g m^{-3} min^{-1});

$\forall = 990\ m^3$ is the volume of the burn room; and

Q is the infiltration rate ($m^3\ min^{-1}$) of outside air into the burn room.

$C(t)$, i.e. the solution to Equation (S1) was obtained from online SMPS measurements, thus Equation (S1) could be solved inversely to obtain Q and then $\dot{E}$.

In the absence of emissions ($\dot{E}$), the solution of Equation S1 is a first-order exponential decay with an e-folding time of $\tau = \forall/Q$. Therefore, Q can be obtained from applying an exponential decay fit to the SMPS measurements after completion of the burn (i.e. when $\dot{E} = 0$), which was confirmed using thermal imaging as described in Section 2.2.2 in the main text.

We then solved Equation (S1) numerically using C(t) as a constraint and with t_0 being the start of the burn to obtain $\dot{E}(t)$. Then using $\dot{E}(t)$, we solved for the particle concentrations corrected for infiltration and wall losses to obtain the correction factor (K) in Equation (5) in the main text as:

$$K = \frac{C_{corrected}}{C_{filter}} \qquad \text{(S2)}$$

Where, $C_{corrected}$ is the corrected concentration and C_{filter} is the actual measured concentration during filter collection (Figure S6).

We note that for experimental permutations that did not involve duff ignition (i.e., all experimental permutation with the exception of BR-Wild), the burn time scales (5-10 minutes) were much smaller than the e-folding time scale of particle decay (> 2 hours). Therefore, the emission and decay processes were largely decoupled, and the corrected particle concentration in the burn room corresponded to the maximum measured concentration after completion of the burn (Figure S6a). For the BR-Wild experiments, which involved duff ignition, the timescales of emission and decay overlapped (Figure S6b).

Figures

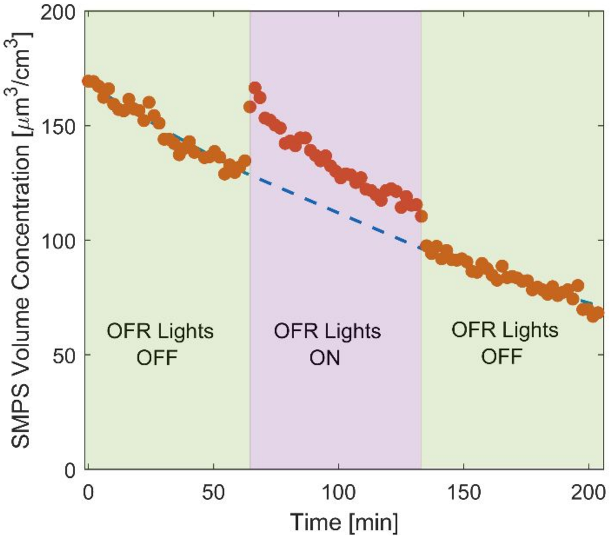

Figure S1. A representative plot (CP-Wild; 11/04/2022) that illustrates the procedure for obtaining organic aerosol (OA) enhancement (Section 2.4 in the main text). The red dots represent aerosol concentrations sampled from the burn room through the oxidation flow reactor (OFR) with the lights off (fresh) and lights on (aged). The blue dotted line is an exponential decay fit to account for particle losses in the burn room. The difference between the measurements and decay fit in the lights on period was used to calculate OA enhancement.

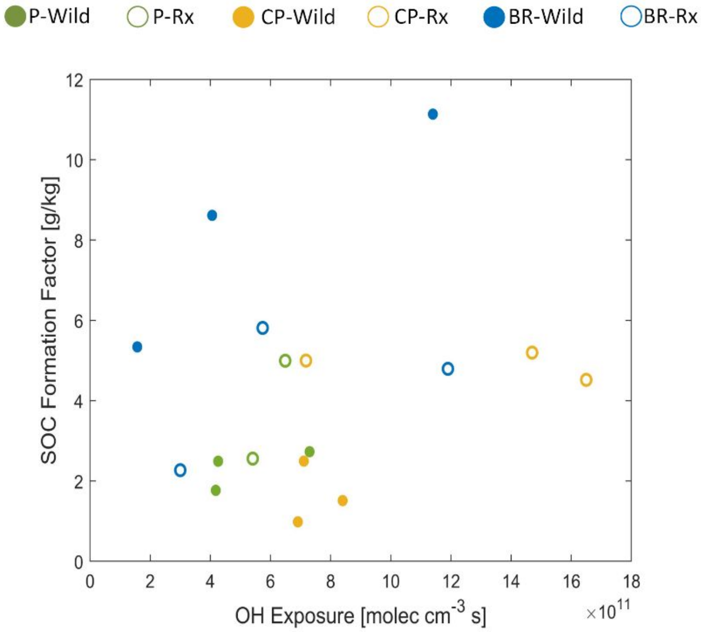

Figure S2. Secondary organic carbon (SOC) formation factors as a function of OH exposure.

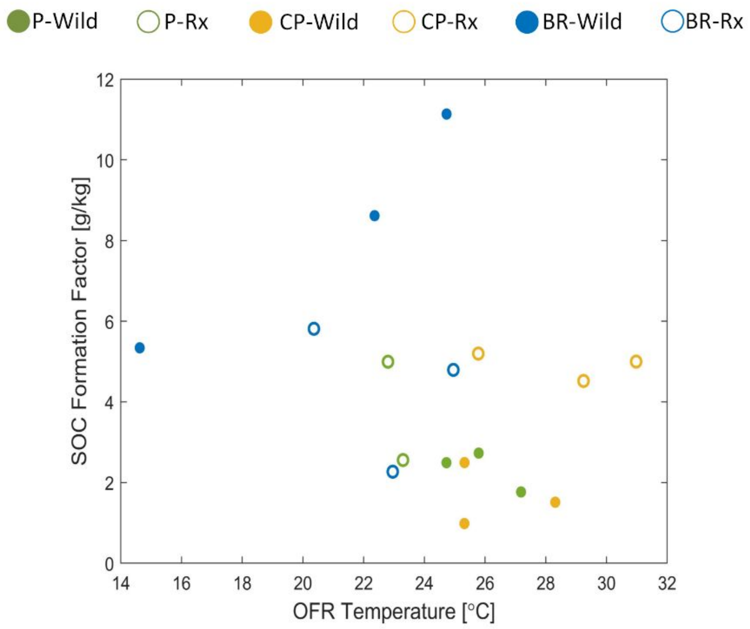

Figure S3. Secondary organic carbon (SOC) formation factors as a function of OFR temperature.

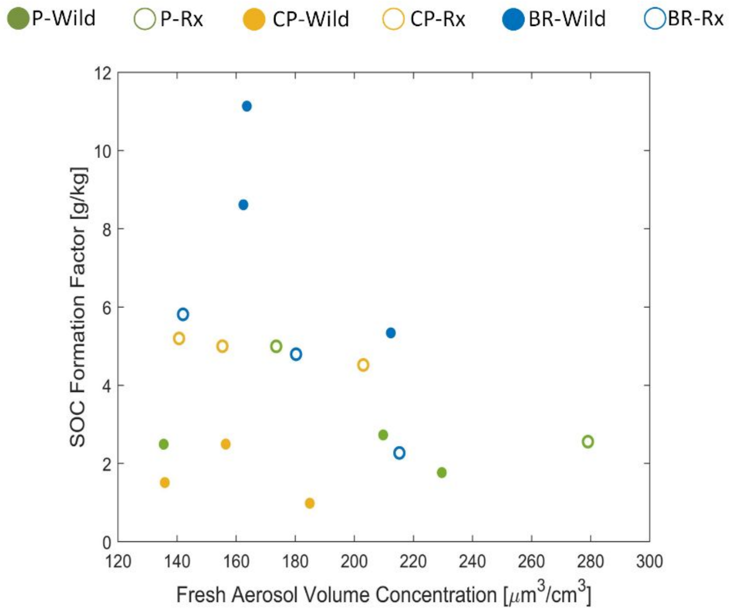

Figure S4. Secondary organic carbon (SOC) formation factors as a function of fresh aerosol volume concentration.

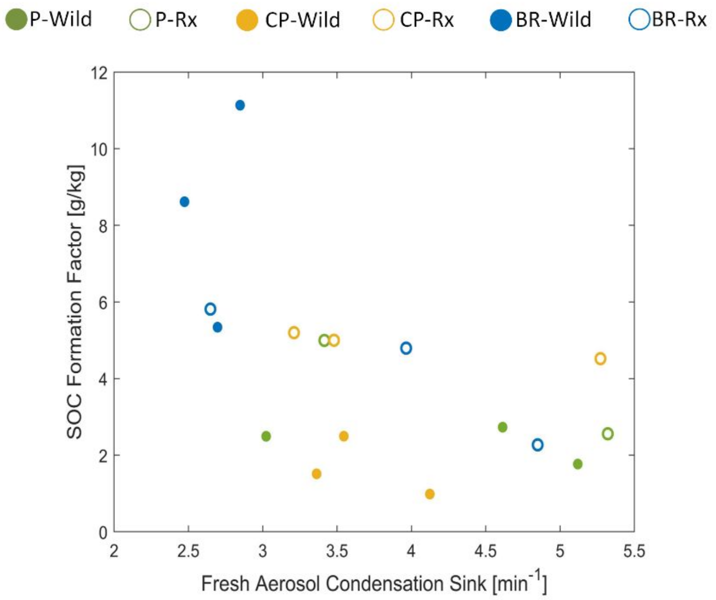

Figure S5. Secondary organic carbon (SOC) formation factors as a function of fresh aerosol condensation sink.

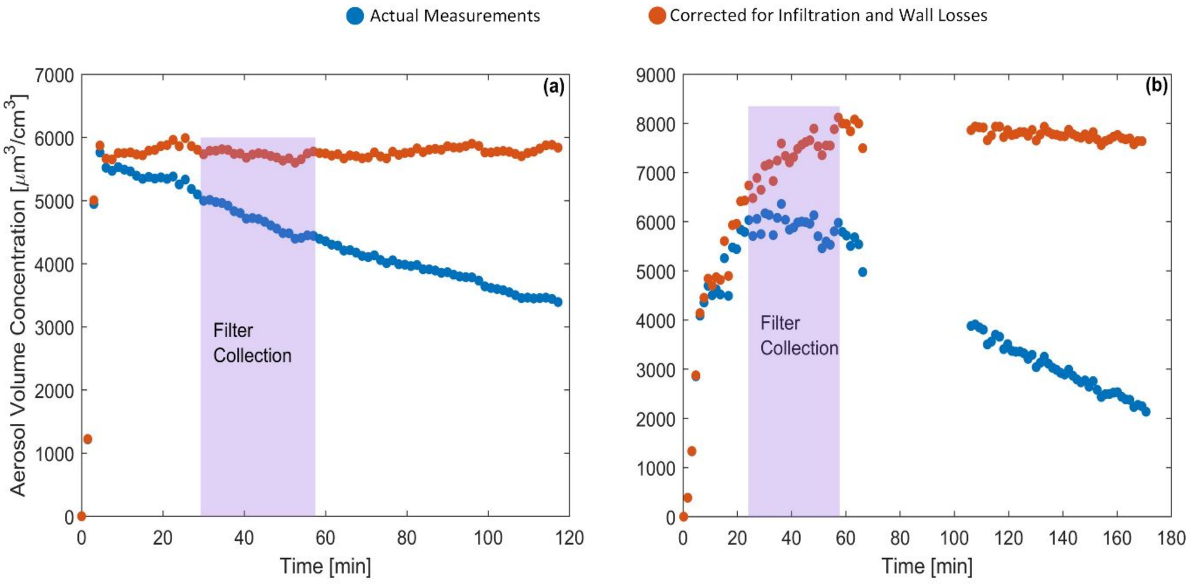

Figure S6. Actual and corrected aerosol concentrations obtained from SMPS representative of (a) a burn that did not involve duff ignition (P-Rx; 10/31/2022) and (b) a burn that involved duff ignition (BR-Wild day; 11/09/2022). The shaded areas correspond to the periods of filter collection. The gap in SMPS data in (b) corresponds to a period when the SMPS was being utilized for size-selection for another set of measurement.

Tables

Table S1. Parameters associated with OFR aging experiments

Date	Experiment	Water Vapor Concentration [g/m^3]	OH Exposure [molec cm^{-3} s]	OFR Temperature [°C]	Fresh Aerosol Volume Concentration [μm^3/cm^3]	Fresh Aerosol Condensation Sink [min^{-1}]
10/25/2022	P-Wild	9.3	4.18E+11	27.2	229.6	5.1
10/27/2022	P-Wild	8.35	4.26E+11	24.7	135.4	3.0
11/1/2022	P-Wild	10.81	7.30E+11	25.8	209.7	4.6
10/28/2022	P-Rx	8.49	5.41E+11	23.3	279.1	5.3
10/31/2022	P-Rx	11.73	6.49E+11	22.8	173.5	3.4
11/2/2022	CP-Wild	10.54	7.11E+11	25.3	156.4	3.5
11/4/2022	CP-Wild	10.31	6.91E+11	25.3	184.9	4.1
11/8/2022	CP-Wild	11.54	8.40E+11	28.3	135.8	3.4
11/16/2022	CP-Wild	2.28	N/A	N/A	N/A	N/A
11/3/2022	CP-Rx	10.54	1.47E+12	25.8	140.6	3.2
11/6/2022	CP-Rx	16.06	1.65E+12	29.2	203.0	5.3
11/7/2022	CP-Rx	15.93	7.18E+11	31.0	155.3	3.5
11/18/2022	CP-Rx	1.87	N/A	N/A	N/A	N/A
11/9/2022	BR-Wild	7.6	4.06E+11	22.4	162.4	2.5
11/12/2022	BR-Wild	12.96	1.14E+12	24.7	163.6	2.8
11/14/2022	BR-Wild	5.02	1.57E+11	14.6	212.3	2.7
11/17/2022	BR-Wild	1.18	N/A	N/A	N/A	N/A
11/10/2022	BR-Rx	9.43	5.74E+11	20.4	142.0	2.6
11/11/2022	BR-Rx	13.9	1.19E+12	25.0	180.3	4.0
11/15/2022	BR-Rx	6.84	3.00E+11	23.0	215.2	4.8

Table S2. Numerical values for the data points in Figure 2.

Date	Experiment	FRE FLIR [MJ]	FRE Telops [MJ]
10/27/2022	P-Wild	0.797	0.973
11/1/2022	P-Wild	0.513	0.641
10/28/2022	P-Rx	0.415	0.576
10/31/2022	P-Rx	0.399	0.498
11/2/2022	CP-Wild	0.757	1.03
11/4/2022	CP-Wild	0.697	0.888
11/8/2022	CP-Wild	0.617	0.78
11/16/2022	CP-Wild	0.582	0.793
11/3/2022	CP-Rx	0.388	0.538
11/6/2022	CP-Rx	0.345	0.464
11/7/2022	CP-Rx	0.384	0.568
11/18/2022	CP-Rx	0.489	0.752

11/9/2022	BR-Wild	1.556	2.53
11/12/2022	BR-Wild	1.402	2.42
11/14/2022	BR-Wild	1.668	2.43
11/17/2022	BR-Wild	1.325	1.87
11/10/2022	BR-Rx	0.152	0.201
11/11/2022	BR-Rx	0.137	0.186
11/15/2022	BR-Rx	0.132	0.189

Table S3. Numerical values for the data points in Figure 3.

Date	Experiment	Fuel Consumption [kg]	FLIR FRE [MJ]	Telops FRE [MJ]
10/25/2022	P-Wild	0.39	0.768	N/A
10/27/2022	P-Wild	0.36	0.797	0.973
11/1/2022	P-Wild	0.29	0.513	0.641
10/28/2022	P-Rx	0.21	0.415	0.576
10/31/2022	P-Rx	0.23	0.399	0.498
11/2/2022	CP-Wild	0.39	0.757	1.03
11/4/2022	CP-Wild	0.36	0.697	0.888
11/8/2022	CP-Wild	0.35	0.617	0.78
11/16/2022	CP-Wild	0.38	0.582	0.793
11/3/2022	CP-Rx	0.26	0.388	0.538
11/6/2022	CP-Rx	0.22	0.345	0.464
11/7/2022	CP-Rx	0.25	0.384	0.568
11/18/2022	CP-Rx	0.29	0.489	0.752
11/9/2022	BR-Wild	1.24	1.556	2.53
11/12/2022	BR-Wild	1.22	1.402	2.42
11/14/2022	BR-Wild	1.35	1.668	2.43
11/17/2022	BR-Wild	1.07	1.325	1.87
11/10/2022	BR-Rx	0.14	0.152	0.201
11/11/2022	BR-Rx	0.12	0.137	0.186
11/15/2022	BR-Rx	0.13	0.132	0.189

Table S4. Numerical values for the data points in Figure 4.

Date	Experiment	FRE FLIR [MJ]	FRE Telops [MJ]	OC [g]	EC [g]	SOC [g]
10/25/2022	P-Wild	0.768	N/A	2.57	0.27	0.69
10/27/2022	P-Wild	0.797	0.858	2.54	0.17	0.91
11/1/2022	P-Wild	0.513	0.513	2.59	0.16	0.80
10/28/2022	P-Rx	0.415	0.475	3.82	0.11	0.55
10/31/2022	P-Rx	0.399	0.416	3.18	0.11	1.13
11/2/2022	CP-Wild	0.757	0.829	2.67	0.25	0.97
11/4/2022	CP-Wild	0.697	0.747	1.99	0.29	0.35

11/8/2022	CP-Wild	0.617	0.663	1.96	0.31	0.53
11/16/2022	CP-Wild	0.582	0.546	2.72	0.17	N/A
11/3/2022	CP-Rx	0.388	0.430	2.75	0.14	1.33
11/6/2022	CP-Rx	0.345	0.383	2.52	0.10	1.01
11/7/2022	CP-Rx	0.384	0.479	3.85	0.12	1.26
11/18/2022	CP-Rx	0.489	0.466	3.88	0.18	N/A
11/9/2022	BR-Wild	1.556	1.812	7.74	0.11	10.71
11/12/2022	BR-Wild	1.402	1.553	10.59	0.17	13.59
11/14/2022	BR-Wild	1.668	1.669	13.85	0.19	7.22
11/17/2022	BR-Wild	1.325	0.840	5.85	0.17	N/A
11/10/2022	BR-Rx	0.152	0.152	1.05	0.08	0.79
11/11/2022	BR-Rx	0.137	0.139	1.32	0.06	0.59
11/15/2022	BR-Rx	0.132	0.132	1.37	0.06	0.30

Table S5. Numerical values for the data points in Figure 6.

Date	Experiment	EF_{EC} [g/kg]	EF_{OC} [g/kg]	FF_{SOC} [g/kg]
10/25/2022	P-Wild	0.68	6.60	1.77
10/27/2022	P-Wild	0.48	6.96	2.49
11/1/2022	P-Wild	0.53	8.74	2.73
10/28/2022	P-Rx	0.42	14.81	2.56
10/31/2022	P-Rx	0.4	11.88	4.99
11/2/2022	CP-Wild	0.63	6.85	2.49
11/4/2022	CP-Wild	0.81	5.49	0.98
11/8/2022	CP-Wild	0.86	5.54	1.51
11/16/2022	CP-Wild	0.44	7.08	N/A
11/3/2022	CP-Rx	0.47	9.32	5.20
11/6/2022	CP-Rx	0.36	9.43	4.52
11/7/2022	CP-Rx	0.43	13.22	5.00
11/18/2022	CP-Rx	0.55	12.07	N/A
11/9/2022	BR-Wild	0.097	6.56	8.61
11/12/2022	BR-Wild	0.16	9.63	11.13
11/14/2022	BR-Wild	0.10	7.51	5.34
11/17/2022	BR-Wild	0.11	3.87	N/A
11/10/2022	BR-Rx	0.61	7.67	5.81
11/11/2022	BR-Rx	0.53	10.77	4.79
11/15/2022	BR-Rx	0.47	10.32	2.27

Table S6. Numerical values for the data points in Figure 7.

Date	Experiment	FLIR FRE_{norm} [MJ/kg]	Telops FRE_{norm} [MJ/kg]	EC/OC

10/25/2022	P-Wild	1.536	N/A	0.103
10/27/2022	P-Wild	1.594	1.944	0.069
11/1/2022	P-Wild	1.026	1.277	0.060
10/28/2022	P-Rx	0.831	1.162	0.029
10/31/2022	P-Rx	0.798	1.002	0.033
11/2/2022	CP-Wild	1.514	2.065	0.093
11/4/2022	CP-Wild	1.394	1.762	0.147
11/8/2022	CP-Wild	1.234	1.548	0.156
11/16/2022	CP-Wild	1.164	1.563	0.062
11/3/2022	CP-Rx	0.776	1.084	0.050
11/6/2022	CP-Rx	0.690	0.943	0.038
11/7/2022	CP-Rx	0.769	1.143	0.032
11/18/2022	CP-Rx	0.978	1.517	0.045
11/9/2022	BR-Wild	0.480	0.781	0.015
11/12/2022	BR-Wild	0.468	0.808	0.016
11/14/2022	BR-Wild	0.562	0.818	0.014
11/17/2022	BR-Wild	0.505	0.713	0.028
11/10/2022	BR-Rx	0.759	1.005	0.080
11/11/2022	BR-Rx	0.685	0.93	0.049
11/15/2022	BR-Rx	0.659	0.945	0.046
